# DNA Methylation Signatures of Response to Conventional Synthetic and Biologic Disease-Modifying Antirheumatic Drugs (DMARDs) in Rheumatoid Arthritis

**DOI:** 10.3390/biomedicines11071987

**Published:** 2023-07-13

**Authors:** Susan Siyu Wang, Myles J. Lewis, Costantino Pitzalis

**Affiliations:** Centre for Experimental Medicine and Rheumatology, William Harvey Research Institute, Queen Mary University of London and Barts Health NIHR BRC & NHS Trust, London EC1M 6BQ, UK; susan.wang@qmul.ac.uk (S.S.W.); myles.lewis@qmul.ac.uk (M.J.L.)

**Keywords:** DNA methylation, rheumatoid arthritis, anti-TNF therapy, biologic therapy, DMARDs, treatment response, precision medicine

## Abstract

Rheumatoid arthritis (RA) is a complex condition that displays heterogeneity in disease severity and response to standard treatments between patients. Failure rates for conventional, target synthetic, and biologic disease-modifying rheumatic drugs (DMARDs) are significant. Although there are models for predicting patient response, they have limited accuracy, require replication/validation, or for samples to be obtained through a synovial biopsy. Thus, currently, there are no prediction methods approved for routine clinical use. Previous research has shown that genetics and environmental factors alone cannot explain the differences in response between patients. Recent studies have demonstrated that deoxyribonucleic acid (DNA) methylation plays an important role in the pathogenesis and disease progression of RA. Importantly, specific DNA methylation profiles associated with response to conventional, target synthetic, and biologic DMARDs have been found in the blood of RA patients and could potentially function as predictive biomarkers. This review will summarize and evaluate the evidence for DNA methylation signatures in treatment response mainly in blood but also learn from the progress made in the diseased tissue in cancer in comparison to RA and autoimmune diseases. We will discuss the benefits and challenges of using DNA methylation signatures as predictive markers and the potential for future progress in this area.

## 1. Introduction

Rheumatoid arthritis (RA) affects around 0.5–1% of the global population. It is characterised by synovial joint inflammation that leads to pain, swelling and reduced mobility and, if poorly responsive to therapy, irreversible damage to cartilage and bone. The aetiology of RA is unknown, but good progress has been made in elucidating the pathogenetic mechanisms. Monozygotic twin studies show a concordance rate of ~12–15% [1,2]. Strong genetic predisposition is associated with HLA-haplotypes specifically the HLA-DRB1 alleles: DR4 and DR1 [3]. Over the last 15 years, genome-wide association studies (GWAS) have also discovered over 100 different loci of RA susceptibility [4]. The overall heritability of the disease is now estimated to be around 66% [5]. Several specific environmental triggers have been associated with the development of RA including exposure to tobacco smoke and organic dust (e.g., silica), obesity and low vitamin D levels [6]. However, contradictory associations have been reported in the literature for some of these risk factors.

In the pathogenesis of RA, the combination of environmental triggers and underlying genetic susceptibility causes the activation of an autoimmune response in the synovium which becomes inflamed, resulting in synovitis. This inflammation is characterized by the activation of immune cells including T-cells, B-cells, macrophages, and the release of pro-inflammatory cytokines, including tumour necrosis factor-alpha (TNF-α), Interleukin-1 (IL-1), Interleukin-6 (IL-6) and Interleukin-17 (IL-17). These cytokines play a pivotal role in perpetuating the inflammatory response and promoting joint damage [7]. TNF-α and IL-1 stimulate osteoclast differentiation causing progressive erosion of bone and cartilage in affected joints [8]. The resulting joint deformities, loss of function, and disability significantly impact the quality of life for individuals with RA [9]. However, the incidence and prevalence of disability have been decreasing in the last 20 years due to the introduction of more effective and aggressive treatment [10].

Although standard therapeutic regimens vary between countries, current first-line therapy consists of conventional synthetic DMARDs (cs-DMARDs), i.e., methotrexate (MTX), sulfasalazine, hydroxychloroquine and leflunomide, as monotherapy or in combination and with corticosteroids if clinically indicated [11]. The most prescribed csDMARD is MTX due to its efficacy and convenient dosing once-weekly regimen. MTX is a folate antagonist but its mechanism of action in RA has not been fully elucidated. It likely induces disease remission in multiple ways including reducing cell proliferation, increasing T-cell apoptosis, downregulating activation of T-cells via adenosine signalling and modulating adhesion molecule expression thereby reducing immune cell migration to the joint [12,13]. 

Second-line therapy consists of biologic DMARDs (bDMARDs), which are monoclonal antibodies to specific key cytokines or cytokine receptors including TNF-α and IL-6, or cellular targets such as CD20 and CD80/86 [11]. Clinical response to treatment is heterogenous, ranging from remission to complete lack of response. ~40–60% of patients treated with MTX experienced a 50% improvement in their joint swelling and pain scores (ACR50) [14] and this is similar for bDMARDs, depending on the specific medication [15,16]. The biological mechanisms behind response are poorly understood and there are no reliable predictors of response in clinical use. 

Clinical parameters including lower disease activity at baseline measured by the Disease Activity Score of 28 joints (DAS28) and not smoking were found to be weakly predictive of treatment response with MTX [17]. Male sex, younger age, and lower baseline DAS28 were associated with treatment response to adalimumab, a TNF-α inhibitor (TNFi) bDMARD, in a large-scale multi-national study [18], whilst current smoking was associated with treatment failure with TNFi [19]. Several studies have shown higher baseline serum CRP levels, and positivity for anti-citrullinated protein antibodies (ACPA) and rheumatoid factor (RF) antibodies were associated with better responses to TNFi, rituximab and tocilizumab [20]. However, other studies have found RF positivity was associated with treatment failure in TNFi [21,22]. Cytokine levels have also been shown to be associated with response. In a study involving 143 RA patients, patients with significant clinical improvement (change DAS28 ≥1.2 at week 16) after starting infliximab (a TNFi) treatment had higher levels of synovial tissue TNF expression compared to those who did not. However, only 10% of the variability in therapy response could be explained by baseline synovial TNF expression [23]. The study of clinical/biochemical biomarkers suffers from discordant results and a lack of independent large-scale validation. More importantly, these biomarkers generally have weak predictive ability, with one study finding that the combined effect of multiple common clinical predictors explains less than 17% of the variation in response [24]. This is likely because genetics, gene expression, and epigenetics play a central role in determining treatment response in RA. 

## 2. Genetic and Transcriptomic Biomarkers of RA Treatment Response

Initial research into markers of MTX response focused on genetic studies with mixed results. Senapati et al., conducted the first genome-wide analysis of MTX treatment response in RA patients. From a cohort of 457 patients, they found 10 new risk loci for poor response using a significant cut-off *p* ≤ 5 × 10^−5^ [25]. In a later genome-wide study of response to MTX in 1424 early RA patients of European ancestry, no single nucleotide polymorphism (SNP) reached genome-wide statistical significance (*p* = 10^−8^) for any outcome measure [26]. The strongest association in this study was rs168201 in NRG3 (*p* = 10^−7^) and this study did not replicate any findings from Senapati et al. Few genetic studies have been conducted specifically for analysing response to sulfasalazine, hydroxychloroquine, or leflunomide. For leflunomide IL-6 -174G/C polymorphism was found to be associated with response. The GG genotype confers a higher risk of therapeutic failure than GC or CC [27]. 

More recently, transcriptomic signatures associated with prognosis and treatment response to csDMARD have been reported. For example, Humby et al., demonstrated that specific gene expression patterns in the synovial tissue at baseline in treatment naïve RA patients can predict the extent of radiographic joint damage after 12 months following csDMARD treatment. Ongoing joint damage is considered an indicator of treatment non-response. The prediction model incorporates rheumatoid factor titre, and the expression level of seven genes using NanoString probes applied to synovial biopsies: SDC1 (encodes plasma cell marker CD138), CSF2 (stimulates growth and differentiation of multiple immune cell lines including granulocytes and macrophages), DENND1C (activates RAB35, which is involved in actin polymerization), CD180 (mediates innate immune response and activates NF-kappa-B), UBASH3A (induces apoptosis in T-cells), CXCL1 (chemoattractant for neutrophils), MMP10 (a matrix metalloproteinase). The predictive algorithm had an area under the curve (AUC) of 0.88 [28], which shows high specificity and sensitivity. 

In a separate analysis of the same cohort of treatment naïve RA patients, Lewis et al., used RNA-Seq to show that expression levels of specific groups of associated genes (gene modules) in the synovial tissue were associated with response to csDMARDs [29]. Specifically, increased expression of the monocyte and chemokine, dendritic cell/antigen-presenting cell, B-cell, and type I IFN signature modules at baseline were associated with larger reductions in DAS-28 CRP score 6 months after csDMARD treatment. Gene expression patterns also changed over time and were associated with response. Modules for CD8+ T-cells, mast cells, and TLR signalling had a significantly higher expression level in EULAR moderate and good responders at 6 months compared to non-responders. Conversely, the CD55+ type 1 fibroblast module had a lower expression in responders [29]. These studies indicate that differences in the synovial gene expression profile of treatment naïve RA patients are associated with variations in treatment response. Lliso Ribera et al., identified in the same cohort a gene set that at baseline could predict the patients that at 12 months required biologic therapy [30].

However, csDMARD treatment in the above studies varied significantly. Patients were treated according to the British Society of Rheumatology’s guidelines and received a diverse range of csDMARD treatments/combinations according to physician and patient preferences. Although these results are promising we are still far from developing a clinically useful test for predicting individual response to specific csDMARDs. 

Research into biomarkers of bDMARD response also initially focused on genetics. Multiple loci and SNPs associated with response to TNFi in RA patients have been identified in recent years, but these findings have not been consistently replicated in different populations. For example, in the PTPRC gene encoding receptor tyrosine-protein phosphatase C or CD45, the rs10919563 G>A polymorphism has been associated with reduced efficacy of the TNFi: adalimumab, etanercept, and infliximab [31]. However, while this was replicated in an independent study [32], a third study failed to find the same association [33]. Another example relates to the TNF promoter SNP G308A (rs1800629) polymorphism, in which in one study of 1040 Caucasian RA patients, the TNF-308AA genotype was significantly associated with poorer response to etanercept [34]. Conversely, the GA genotype correlated with a better response to adalimumab [35]. Polymorphisms in the IL-6 promoter region have been associated with improved response to TNFi in the Spanish population [36,37]. Additionally, polymorphisms on steroid hormone-related genes CYP2C9 and CYP3A4 have also been associated with response [38]. Analysis of three large-scale GWAS studies found 12 loci associated with TNFi response [39,40,41] but this could not be replicated in a similar large-scale study of 755 RA patients [42]. These results demonstrate that the relationship between genotype and treatment response is very complicated, and it is possible that small variations in concert may produce differences in response. Additionally, genotype varies significantly between racial groups, which means there is unlikely to be one specific set of polymorphisms that affects treatment response across the entire human population. 

Transcriptomic signatures associated with bDMARD response have been found [43,44]. In a large-scale, synovial biopsy-driven, randomised control trial of rituximab vs. tocilizumab, patients were classified as B-cell rich or B-cell poor by the expression levels of a specific group of genes related to B-cells. B-cell-poor patients showed a lower CDAI50 response rate to rituximab compared to tocilizumab. B-cell-rich patients had the same response rate to rituximab and tocilizumab [43]. Conversely, classifying patients as B-cell-rich or poor solely based on histology did not show any statistically significant difference in CDAI50 response rate between rituximab and tocilizumab. In a separate analysis of the same cohort of patients, 6625 differentially expressed genes (DEGs) were found between rituximab responders vs. non-responders and 85 DEGs were found between tocilizumab responders vs. non-responders [44]. Genes upregulated in rituximab responders included leukocyte-related genes, macrophage, chemokine and cytokine-related genes and members of the immunoglobulin (Ig) superfamily. Lymphocyte and Ig genes were also upregulated in the synovial tissue of tocilizumab responders. Downregulated genes in rituximab responders and tocilizumab responders were predominately fibroblast-related genes, Hox genes and complement genes. As this was a cross-over trial, it was possible to identify a group of double non-responder individuals who failed both rituximab and tocilizumab during the study. Furthermore, since all individuals who entered the study were anti-TNF non-responders, this group of individuals truly represent a multi-drug resistant or “refractory” group who failed to respond to three biologics. Notably, these patients had significant upregulation of fibroblast and extracellular matrix-encoding genes such as fibroblast growth factor (FGF), homeobox (HOX) and NOTCH family genes, together with multiple cell adhesion molecule and collagen-encoding genes. This suggests that multidrug-resistant RA or persistent non-response is associated with a specific transcriptomic profile which is dominated by fibroblast-related genes rather than classic adaptive immune system-related genes. 

Multiple gene sets have been identified in whole blood, which are associated responses to TNFi including infliximab [45,46] and adalimumab [47] and validation of these gene sets in a separate cohort of patients has been conducted, which showed a sensitivity of 71% and a specificity of 61% in predicting response to TNFi [48]. 

Jak inhibitors (JAKI) are a class of target synthetic DMARD that were first introduced for widespread clinical use by the approval of tofacitinib by the FDA in 2012. Research into JAKI has mainly focused on their efficacy, mechanisms of action and safety profile. Investigations into biomarkers of response to Jak inhibitors lag the other DMARDs due to the relatively shorter period they have been in use. Valli et al., found that tofacitinib treatment reduced both the DAS28 score and the levels of certain circulating pro-inflammatory markers, the most pronounced reduction being in IL-6, C-X-C motif chemokine ligand 1 and matrix metalloproteinase-1. Additionally, higher baseline circulating levels of IL-6 and lower levels of C-C motif chemokine 11 were associated with DAS reduction post-treatment [49]. However, reduction in cytokine levels is known to be a biological function of JAKI and the study did not determine whether the same reduction may also in non-responders as well. Ciechomask et al., found that circulating levels of miRNA-19b-3p are associated with baricitinib response in RA. The levels of this miRNA are higher in RA patients compared to healthy controls and there is a statistically significant reduction in levels 3 months after treatment, which corresponded with a significant DAS28 score reduction [50]. Again, this study does not compare the differences between responders and non-responders. Both studies recruited small cohorts of patients (54 and 44 RA patients, respectively), and their results have not been replicated. 

These results show that there are distinct synovial transcriptomic profiles of both response and non-response to csDMARDs and specific bDMARDs. However, genetic and transcriptomic studies only provide insight into one aspect of the biological mechanisms underlying response. Genetic variation affects epigenetic modifications including DNA methylation, which in turn affects gene transcription. Therefore, DNA methylation studies should help to further elucidate the biological mechanisms of response. Many recent studies have focused on DNA methylation as a biomarker of RA treatment response with a strong, growing body of evidence that DNA methylation plays an important role in both pathogenesis of RA and treatment response.

## 3. An Overview of DNA Methylation

Epigenomics refers to modulations that influence gene expression but do not change the underlying genetic code. These include DNA methylation, histone modification, and microRNA modulation [51]. This review will primarily focus on DNA methylation as it is the most widely studied epigenetic modification. 

**Figure 1:**
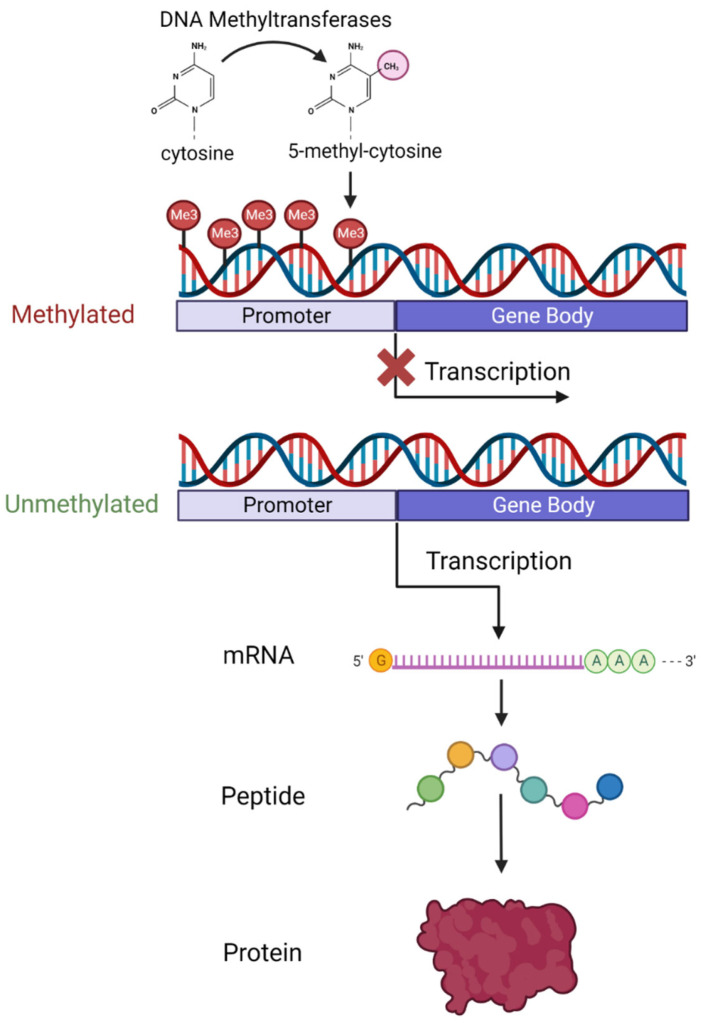
Diagram showing the process of DNA methylation and how methylation at the promoter region of genes affects transcription and ultimately protein expression levels. Created with BioRender.com accessed on 22 May 2023.

As schematically illustrated in Figure 1, DNA methylation is the process whereby a methyl group is added to a cytosine–guanine (C-G) dinucleotide (CpG) by DNA methyltransferase enzymes.

CpG sites exist across the entire genome in both coding and non-coding regions but CpG sites then cluster in groups called islands. A total of 70% of islands are found in promoter regions of genes [52], and 50% of human genes initiate transcription from a CpG site [53]. Methylation in the promoter region appears to interfere with transcription factor interaction with the underlying DNA. Hypermethylation in the promoter region is usually associated with decreased expression of the gene or even transcription silencing [54]. Hypomethylation in contrast is associated with active transcription and increased gene expression [51]. Gene body and intergenic methylation is not associated with transcriptomic silencing and has a more context-dependent relationship that differs between genes [55]. Methylation of the CpG site in the gene body is thought to prevent spurious transcription factor binding and regulate alternative splicing [56]. 

DNA methylation patterns are highly tissue and cell-type-specific, which reflects its essential role in normal development. DNA methylation enables selective temporal activation of lineage-specific genes, and suppression of pluripotency genes in the early embryonic stages, which ensures proper establishment of gene expression patterns for specific tissue and cell type development [57]. DNA methylation is vital in controlling the expression of imprinted genes, enabling allele-specific expression of gene clusters, which are essential for normal development. Parent-specific methylation patterns are introduced during gamete differentiation and maintained throughout life [58]. DNA methylation plays an important role in sex development through inactivation of the second X chromosome in females, and the development of sex-specific tissues/cells. Epigenetic differences may also contribute to differences in disease susceptibility between sexes [59]. Throughout life, DNA methylation remains an essential mechanism for dynamic gene expression regulation in response to external and internal stimuli [60]. Diversity in methylation patterns between individuals is affected by a combination of genetic and environmental factors. Underlying genetic variation including sequence-specific allelic variations influence methylation patterns [61] and many environmental factors including age, early life poverty, stress, diet, smoking, obesity, and diseases affect DNA methylation patterns [61,62].

## 4. DNA Methylation in RA

There has been significant progress in recent years in DNA methylation research in RA showing that the methylome of patients displays distinct differences compared with healthy controls. Liu et al., performed the largest epigenome-wide association study to date on the whole blood samples of 354 ACPA-positive RA cases and 337 controls [63]. Their results identified genome-wide statistically significant differential methylation in nine CpG locations within the MHC region. Additionally, by analysing the interaction between genotype and differential methylation it emerged that methylation at these susceptibility loci is likely to mediate the genetic risk for the development of RA. An independent smaller study of 62 RA patients also found significant methylation differences between RA and healthy controls with methylation of peripheral blood monocytes found to be directly linked with DAS28 score in RA patients through the action of inflammatory cytokines [64].

Recent studies into the methylation pattern of specific immune cell types in RA including fibroblast-like synoviocytes (FLS), monocytes and lymphocytes have also revealed interesting insights into RA pathogenesis. For example, peripheral blood mononuclear cells (PBMCs) of RA patients were found to be globally hypomethylated compared to healthy controls [65], while an epigenome-wide association study found, again in PBMCs, 1046 different DNA methylation sites linked to disease pathogenesis [66]. Furthermore, distinct DNA methylation signatures in peripheral blood B-cells and T-cells are already seen in early RA patients compared with healthy controls [67]. In established RA, important genes involved in RA pathogenesis, which were found to be hypomethylated in the B-cells and distinguish patients from healthy individuals include: BARX2 encodes for a transcription factor, which influences cell processes involved in cell adhesion and migration, ASB1 mediates degradation of proteins including JAK2, involved in RA inflammation, ADAMTS17 a metalloprotease, MGMT a methyltransferase [68]. 

DNA methylation patterns in the cluster of differentiation 4 positive (CD4+) T-cells of RA patients are different compared to healthy controls. In RA patients, *JUN*, *STAT1*, *PTEN*, and *CD44* genes exhibit hypermethylation, while *KRAS* and *ALB* show hypomethylation. Gene ontology (GO) enrichment studies indicate that the differentially methylated genes in RA are connected to T-cell biological processes, suggesting that DNA methylation plays a role in regulating CD4+ T-cell function in RA [69]. Comparisons between memory CD4+ T-cells and naive CD4+ T-cells in RA patients reveal an increased number of differentially methylated positions (DMPs) in memory cells. Most of these DMPs exhibit increased DNA methylation in RA patients with active disease. Specifically, differential hypomethylation of *UBASH3A*, a gene involved in antigen presentation to T-cells was found [70]. T-cells exhibit cellular heterogeneity and can differentiate into subsets. In RA, T-helper (Th) 1 and Th17 subsets contribute to inflammation, while Th2 cells can inhibit Th1 and Th17 cell function and dampen inflammation. DNA methylation sites in CD4+ naïve T-cells and memory CD4+ T-cells of RA patients indicate a shift towards Th17 cell development and there is differential hypomethylation of IFN-related genes increasing gene expression, which serves to perpetuate chronic inflammation [71,72]. Aberrant DNA methylation in T-cells of RA patients has also been reported to cause T regulatory (Treg) and Th17 imbalances leading to the amplification of inflammation, which is resolved by MTX treatment [73]. Cribbs et al., found hypermethylation in the NFAT binding site of the *CTLA-4* promoter region leading to reduced production of CTLA-4, which was associated with compromised Treg activity in RA [74]. 

Studies of FLS in RA patients showed significant differential methylation compared with osteoarthritis (OA) patients [75]. Nakano et al., found 1859 DMPs between RA and OA patients with hypomethylation of CpG sites located on key genes in RA pathogenesis including *CASP1*—encodes Caspase 1 that induces cell apoptosis, *MAP3K5* a MAP kinase involved in the innate immune system, *STAT3* a transcription activator, activated in response to cytokine signalling, *MEFV* (pyrin) that generates inflammation in response to interferon-gamma signalling. Hypomethylated CpG sites were primarily located on gene pathways involved in cell adhesion/migration and extracellular matrix interactions [76], also supported by two additional studies into FLS [77,78]. Interestingly, the latter study also found that global DNA methylation patterns were also joint-specific in RA and OA FLS, providing a plausible explanation as to why RA has a propensity to attack certain joints [78]. The DMPs discovered in FLS of RA patients also showed some overlap (~20%) with DMPs found in CD4+ naïve T-cells in the peripheral blood of RA patients [79], suggesting a possible common DNA methylation change linked to RA pathogenesis. Subsequent research found the global hypomethylation in RA-FLS is likely caused by the downregulation of DNMT1 (DNA methyltransferase 1) and DNMT3A, which are important enzymes involved in DNA methylation, driven by the inflammatory environmental [80]. DNA methylation of the *PTEN* gene promoter region has also been shown to activate FLS in RA pathogenesis [81]. These studies show that the methylome of RA patients differs significantly from healthy controls and likely reflects the pathogenesis of RA.

Analysis of immune cells from the blood and synovium of RA and OA patients showed the largest differences in methylation were between different tissues, rather than between disease states [82]. This suggests the methylome of immune cells is different in the synovium versus the blood and it is likely that the synovium methylome more accurately reflects the biological mechanisms of pathogenesis in RA. 

Single-cell RNA sequencing analysis of cells derived from synovial tissue has found distinct subpopulations of inflammatory cells and fibroblasts that are not present in the blood [83]. Within the CD4+ T helper cell population, a distinct subset marked by high expression levels of MAF (transcription factor), CXCL13 (B lymphocyte chemoattractant), and PDCD1 (immune-inhibitory checkpoint receptor PD-1) was detected, which had not been identified in previous single-cell RNA sequencing studies of human PBMCs. Within Natural Killer cells, a subpopulation expressing high levels of cytokines XCL1 (lymphotactin) and XCL2 was discovered. These cytokines regulate fibroblast production of matrix metalloproteinases and direct lymphocyte migration in synovial tissue. Analysis of fibroblasts found two transcriptomically distinct fibroblast subsets that have distinct anatomic locations within the synovium. This study shows that the synovium has a unique, diverse range of previously unknown cell subpopulations with distinct transcriptomic signatures, which likely contribute to the pathogenesis of RA and can affect response to treatment. 

Histological studies of immune cells in the synovial tissue of treatment naïve early RA patients found three distinct pathotypes: a lympho-myeloid type dominated by the presence of B-cells and myeloid cells, a diffuse-myeloid type myeloid cells but very few B-cells and the pauci-immune type characterised by scanty immune cells and prevalent stromal cells [29]. The lympho-myeloid and myeloid pathotypes are associated with higher disease activity and acute phase reactants, but a better overall response to conventional RA treatment [28]. Each synovial pathotype had a distinct transcriptomic profile and specific gene expression signatures are associated with treatment response [29]. 

DNA methylation analysis of synovial tissue is still in its infancy but, as the methylation process is highly cell-specific, the methylation status of synovial tissue will naturally reflect its diverse cellular composition as per the above-described pathotypes. Consequently, each pathotype is likely to have a distinct DNA methylation profile and it would be interesting to establish whether specific synovial DNA methylation signatures will enhance our future ability to predict prognosis and treatment response in RA.

## 5. DNA Methylation Biomarkers in Other Autoimmune Diseases

Research into DNA methylation in other autoimmune inflammatory diseases is still in the early stages but there have been some promising results. A systematic review of methylation in inflammatory bowel disease found consistent differential methylation was identified for 256 DMPs in the peripheral blood of IBD patients compared to healthy controls [84]. DNA methylation in the whole blood can also be used to differentiate Crohn’s disease from intestinal tuberculosis, which is difficult to achieve clinically [85]. These studies suggest DNA methylation could provide biomarkers for future non-invasive diagnosis. In systemic lupus erythematosus (SLE), DNA methylation has been used to identify disease subtypes [86] and methylation signatures related to prognosis and treatment response have been found [87], suggesting a future role for DNA methylation in diagnosis and treatment allocation. 

## 6. DNA Methylation as a Biomarker 

DNA methylation has great potential as a biomarker because it is dynamic and constantly modified in response to stimuli. It is more stable than gene expression at transcript and protein levels and is inherited between cell divisions [54]. The most successful use of DNA methylation signatures as biomarkers is in the diagnosis and treatment of cancer. Tumour cells have highly aberrant and unique DNA methylation patterns that differentiate them from normal cells. Diagnostic DNA methylation tests for early detection of cancers have been developed and show some promise. PanSeer panel detects circulating tumour methylated DNA (ctDNA) that matches 595 specific high-risk locations on the genome. The panel has high sensitivity (88%) and specificity (96%) in detecting five common cancer types from peripheral blood samples of asymptomatic patients up to four years before conventional diagnosis [88]. It is in the advanced stages of development into a clinical test though it has not been approved by the Federal Drug Administration (FDA) or the National Health Service (NHS) for clinical use yet. Two DNA methylation-based diagnostic biomarkers have been approved by the FDA for clinical use in the diagnosis of colorectal cancer [89] and DNA methylation at baseline has been shown to be a predictive response to therapy in colorectal cancer [89,90]. DNA methylation patterns have been shown to predict response to neoadjuvant therapy in specific types of breast cancer [91,92].

However, using DNA methylation as a biomarker also poses certain challenges. Age, sex, smoking, alcohol consumption, diet, stress, and exposure to environmental chemicals can all induce changes in DNA methylation patterns [62]. These confounding factors must be controlled for when analysing DNA methylation as biomarkers in diverse patient population groups. DNA methylation is highly cell-type-specific and making direct comparisons between studies investigating different cell types or tissues can be very difficult. This prevents high-quality meta-analyses and limits statistical power. In studies of heterogenous samples such as whole blood the methylation signature is a summation of all the cell types. This is challenging to correct in post hoc analysis despite the existence of targeted algorithms [93]. Therefore, DNA methylation signatures can be very useful biomarkers, but discovery requires overcoming the unique challenges posed by its biology.

## 7. DNA Methylation and Response to csDMARDs

As previously discussed, genetic and transcriptomic signatures linked with treatment response in RA have been found. As gene expression is regulated by DNA methylation, it is possible that DNA methylation signatures linked to csDMARD response also exist and might provide a more accurate biomarker as DNA methylation is more stable than gene expression levels. 

So far, all DNA methylation studies exploring associations/predictability of response have been performed on whole blood or cells isolated from blood, as this is the most accessible form of tissue for investigation. Most studies investigating the relationship between DNA methylation and csDMARD response centre on MTX as a monotherapy or in combination with steroids and other csDMARDs (Table 1). Recent studies have focused on how MTX changes the methylome both globally and in specific cell lines, and whether differences in baseline DNA methylation patterns are related to response.

In brief, MTX treatment has been shown to change global methylation patterns in important immune cells. De Andres et al., found treatment naïve RA patients have global hypomethylation of blood T-cells and monocytes compared to healthy controls [94]. Quantitative PCR showed a corresponding decrease in the expression of DNA methyltransferase 1 (DNMT1), the enzyme that maintains DNA methylation patterns, in both cell types. Increased expression of enzymes involved in demethylation was also found in monocytes. In MTX-treated patients, global DNA methylation levels were the same as in healthy controls, indicating that treatment reversed the global hypomethylation [94]. The treated patients included in this study had a mean DAS28 score of 1.6 which is considered disease remission, suggesting that reversal of global hypomethylation in T-cells and monocytes may be a good indicator of disease control. These findings are supported by Liebold et al., who showed global hypomethylation in peripheral blood monocytes (PBMCs) of treatment naïve RA patients compared to controls [65]. Guderud et al., compared MTX-treated patients in remission with healthy controls and found 80% of DMPs in MTX-treated patients were hypermethylated in CD4+ memory T-cells but the proportion of hyper- versus hypomethylated sites was equivalent in CD4+ naïve T-cells [70]. A second study carried out by the same team compared DNA methylation patterns before and three or six months after initiating MTX treatment in the same group of patients. Treatment was associated with 226 significant DMPs in CD4+ naïve T-cells of which 63% were hypomethylated post-treatment, and 188 DMPs in CD4+ memory T-cells with 59% displaying hypomethylation [100]. The discrepancies between the above studies, especially in relation to the degree of hypomethylation, are likely due to the different cell types investigated, methodological differences, small sample sizes, and differences in treatment regime (MTX monotherapy versus combination therapy). These data indicate that MTX treatment generally reverses global hypomethylation in immune cells, but the exact changes are specific to the cell type and likely reflect the diverse mechanisms of action attributed to MTX. 

Global methylation patterns have been shown to be correlated with clinical response. Gosselt et al., investigated the global methylation of leukocytes from 181 RA patients treated with MTX or MTX and 2 other csDMARD. They discovered that higher baseline global DNA methylation was associated with smaller decreases in DAS28 CRP from baseline and with MTX non-response after 3 months of treatment [96]. Liebold et al., analysed global DNA methylation patterns of PBMCs and lymphocytes in 45 RA patients before and three months after starting treatment with either MTX, sarilumab, tofacitinib or baricitinib. The results demonstrated a strong negative correlation between methylation levels and DAS28-ESR scores at both time points [65]. Comparing methylation levels to individual components of the DAS score showed a strong correlation with swollen and tender joints, but there was no correlation with other parameters including ESR, CRP, or VAS. Although it is not possible to elucidate the individual effect of MTX from this study, it may indicate global hypermethylation in inflammatory cells at baseline is associated with response to RA treatment. 

DNA methylation at specific CpG sites has been linked to csDMARD response. Glossop et al., extracted peripheral blood samples from 46 cs-DMARD naïve RA patients, who were subsequently treated with MTX, hydroxychloroquine, or sulfasalazine. Genome-wide DNA methylation profiling of T-cells found six statistically significant DMPs between responders and non-responders after FDR correction. Two specific CpG sites located on the genes: *ADAMTSL2* (encodes a secreted glycoprotein that interacts with the extracellular matrix) and *BTN3A2* (gene located in the MHC class 1 locus, encoded protein inhibits the release of IFN-gamma from activated T-cells) were very strongly associated with treatment response. Increased methylation at these sites in combination was the biggest predictor of response (80.0% sensitivity, 90.9% specificity) [97]. Nair et al., investigated DNA methylation in whole blood collected from 72 RA patients before and 4 weeks into MTX treatment but found no differential methylation in baseline samples between EULAR good and poor responders at 6 months [98]. However, two CpG sites showed significant methylation changes at 4 weeks associated with clinical response status by 6 months, though the significant cut-off used was a nominal *p* value of 1 × 10^−6^ instead of standard FDR correction. Four additional DMPs at the 4-week time point predicted an improvement of swollen joint count and CRP at 6 months. Gosselt et al., found no DMPs or differentially methylated regions (DMRs) at genome-wide significance level in pre-treatment PBMCs between 68 responders and non-responders to MTX monotherapy or combination therapy [99]. The discrepancies between these studies are likely due to the different cell types investigated. Glossop et al., only analysed T-cells whereas Nair et al., analysed whole blood, which is a heterogenous mixture that may mask methylation changes in specific types of immune cells. 

Although the results from these studies are interesting, there is no consensus on DNA methylation patterns of csDMARD response and more research needs to be conducted to find a reliable methylation biomarker of response. Additionally, most studies look at a combination of different csDMARDs so it can be difficult to understand the DNA methylation patterns associated with response to specific agents. Monotherapy studies primarily focus on MTX and to date, there are no specific DNA methylation studies of response to monotherapy with sulfasalazine, leflunomide, or hydroxychloroquine, most likely because there are relatively few patients on monotherapy with these agents. 

DNA methylation analysis of synovial tissue is a more promising future avenue of investigation compared to blood. Transcriptomic signatures of response have been found in the synovial tissue [29], and therefore DNA methylation signatures of response may also be found.

## 8. DNA Methylation and Response to Biological and Targeted Synthetic (Jak-Inhibitors-JAKI) DMARDs

Research into DNA methylation patterns associated with biologic response is still at a relatively early stage, with only a handful of studies published confined to the peripheral blood (Table 2). To date, no specific studies investigating DNA methylation biomarkers of JAKI response have been carried out. Liebold et al., investigated global hypomethylation in the PBMCs of a mixture of patients treated with either MTX, sarilumab, tofacitinib, or baricitinib. It is impossible to separate the results to ascertain the effect of methylation on response to just the JAKI [65].

Specific DNA methylation signatures of response have been identified by Plant et al., in an epigenome-wide association study on pre-treatment whole blood samples from 72 etanercept-treated RA patients [101]. Five CpG sites were found to be significantly differentially methylated at baseline between responder groups with a false discovery rate of <5%. The top two DMPs are mapped to exon 7 of the *LRPAP1* gene on chromosome 4. This gene encodes a protein that interacts with the low-density lipoprotein (LDL) receptor-related protein and facilitates its proper folding and localization. It is not known to be involved in immune response or inflammation and the exact role of methylation at this locus in RA is still unclear. Methylation quantitative trait loci analysis was carried out on the LRPAP1 loci. The A allele of rs3468 SNP correlated with higher methylation levels at the top two DMPs and increased risk of poor response. SNP rs3468 was analysed in an independent cohort of 1,204 TNFi-treated RA patients and each stepwise increase in the A allele from GG to AA caused a 1.28-fold increased risk of being in the poor response group. This suggests genotype affects DNA methylation at key positions, and the combination of these factors influences individual treatment response. However, a longitudinal analysis of RA patients treated with TNFi conducted by Julia et al., found no genome-wide significant DMPs at baseline between responders and non-responders [102]. This study found that TNFi significantly changed the whole blood methylome over 3 months in all patients. Methylation patterns in post-treatment samples more closely resembled that of healthy individuals. These changes were found equally in both responders and non-responders suggesting that TNFi changes the methylome irrespective of response [102]. The discrepancy between these two studies could be due to the different DNA methylation arrays used. Although both studies used Illumina arrays, Julia et al., performed their study on the EPIC array which has >850,000 probe sites, the majority of which are in the inter-genomic region (IGR), whereas Plant et al., used the 450K array which has around 450,000 probes situated mainly on promoter regions and the gene body. These two arrays have a significant overlap [104] and in theory results from these arrays are directly comparable. However, the increased number of probes from the EPIC array means that in statistical testing the *p* value threshold to pass FDR also increases and therefore probes that were statistically significant from the 450K array may not be significant when tested with all the probes from the EPIC array. 

Tao et al., investigated gene expression and DNA methylation patterns associated with etanercept and adalimumab response [103]. Differential gene expression in PBMCs was found between response groups for TNFi, but the differentially expressed genes (DEGs) showed very little overlap (<2%) indicating response is defined by distinct gene signatures for each medication. Epigenome-wide association study in PBMCs found nominally significant (*p* < 0.05) DMPs between response groups but none at the genome-wide significance level. Globally more hypermethylated DMPs were found in etanercept responders compared to adalimumab responders, suggesting there is also a distinct DNA methylation pattern of response for each drug. Using gene expression data from PBMCs, monocytes and T-cells and the DNA methylation data from PBMCs, the investigators built a predictive algorithm using machine learning. The model for predicting etanercept response using DNA methylation had an overall accuracy of 88%, which surpassed the accuracy of pure gene expression models (73% to 79%). The adalimumab DNA methylation model had a high overall accuracy of 84%, which was similar to the pure gene expression model using DEGs from PBMCs (85%). This suggests that DNA methylation patterns may be used to generate accurate predictive algorithms for TNFi response. Further research into integrating DNA methylation, transcriptomic and genotype data into one predictive model may produce more accurate algorithms. 

## 9. Discussion

DNA methylation signatures have shown great promise as biomarkers for diagnosis and predicting treatment of cancer. In contrast, DNA methylation research in RA is still in the early stages and no reliable biomarkers of treatment response have been found. The major challenge facing this area of research is the relatively small methylation differences between treatment groups in RA. Methylation variations of <5% are the norm for non-cancerous tissue [105] and large sample sizes are required to provide sufficient power to detect the subtle differential methylation. Mansell et al., estimate that using the Illumina Human Methylation EPIC array, a minimum of 200 samples are required to provide 80% power to detect a 5% mean methylation difference at 80% of CpG sites and to detect a mean methylation difference of 2%, 1000 samples are required [105]. Therefore, nearly all DNA methylation studies in RA were statistically underpowered for detecting differences in DNA methylation of <5% between groups. This explains why many RA studies could not find statistically significant results with epigenome-wide association studies [99,102], and significant findings in other studies have not been replicated. Meta-analyses for methylation studies of treatment response are very difficult to perform due to the lack of standardisation between studies. As shown in Table 1, treatment regimens used in csDMARD studies vary significantly, with some studies using data from patients treated with two or more regimes [96,97,99]. Additionally, DNA methylation is highly cell-specific [52] and it is not possible to directly compare results from studies analysing different cell types. Methods for measuring DNA methylation also vary greatly and studies with different methods cannot be directly compared. Global methylation assessments use either mass spectrometry or immunofluorescence-centred methods [65,94,96], whereas methylation microarrays allow for analysis of methylation levels at specific CpG sites across the genome [98,99]. 

Further challenges facing methylation research in RA treatment response relate to the lack of samples from the diseased tissue, i.e., the synovium. All DNA methylation studies of treatment response to date have used peripheral blood samples but the blood methylome is affected by many different factors, including other causes of inflammation independent of RA. In contrast, the methylation patterns of the synovium and synovium-derived cells may more accurately reflect the biological mechanisms underlying RA-specific inflammation.

## 10. Future Perspectives

DNA methylation analysis of the whole synovial tissue and/or single cells derived from synovial tissue digestion is the logical next step and is more likely to provide specific biomarkers for RA treatment response in the future. A study comparing gene expression (RNA-seq) in the synovium versus the blood of RA patients showed much greater differential gene expression in the synovium compared to the blood [29]. In that study, three different RA subtypes linked to distinct gene signatures were described but these differences were not present in the blood [29]. Most importantly, synovial transcriptomic signatures of response to both csDMARDs and bDMARDs have already been reported [29,43,44]. Therefore, analysis of DNA methylation of synovial tissue has the potential to yield similarly useful biomarkers of treatment response. 

Methods for generating DNA methylation data have progressed significantly. The Illumina BeadChip arrays—450K and EPIC, are relatively new but increasingly used for epigenome-wide association studies [106]. These arrays provide a high number of probe sites, 450,000 and 850,000, respectively. Although they do not include all the known dynamically regulated CpG sites [107] and do not cover the whole genome, they provide a relatively fast and cost-effective method to generate detailed DNA methylation data from large groups of samples. There is a significant overlap between the 450K and EPIC arrays [104] and methylation measurements correlated well [108]. This allows for a direct comparison of results from studies using either of the two arrays and easier replication of previous findings. In the future, as more research using these arrays is published, it will be possible to perform accurate meta-analyses with sufficient power to detect small methylation differences between treatment response groups. 

Finally, DNA methylation should not be viewed in isolation as it is only one type of epigenetic modification. Histone modifications and microRNAs also contribute to the pathogenesis of RA, in part through their regulation of DNA methylation [109]. Further research is needed to elucidate the complex role epigenetics plays in RA as part of the multi-omic regulatory landscape in conjunction with genomics, transcriptomics, and proteomics. An integrated multi-omic approach is likely to better elucidate the biological mechanisms underlying pathogenesis and treatment response in this complex condition. This combined approach has already been successfully used in tumour profiling [110] and in predicting treatment to immunotherapy in certain cancers [111]. A similar integrated multi-omic method will likely be the most fruitful course of future research to identify specific biomarkers and build predictive models of treatment response in RA.

## 11. Conclusions

In conclusion, the emerging evidence from numerous studies highlights the potential of DNA methylation as a promising biomarker for predicting the response to treatment in patients with RA. DNA methylation alterations have been observed in key genes and regulatory regions associated with immune system dysregulation and inflammatory pathways in RA. These epigenetic modifications, particularly in genes involved in B-cell/T-cell differentiation and cytokine signalling, appear to play a crucial role in pathogenesis and treatment response.

Although substantial progress has been made, further research is required to validate the utility of DNA methylation as a reliable biomarker of treatment response in RA. Large-scale prospective studies, including diverse patient populations and different treatment regimens, are warranted to establish robust associations and refine predictive models. Additionally, investigating the dynamic nature of DNA methylation patterns throughout the course of treatment will provide valuable insights into the underlying mechanisms of therapeutic efficacy. 

DNA methylation holds significant promise as a biomarker for predicting treatment response in RA. Its potential to provide valuable insights into disease mechanisms and guide personalized therapeutic approaches makes it an exciting area of research in the field of rheumatology. Future advancements in our understanding of DNA methylation dynamics and its functional consequences will undoubtedly contribute to improved patient outcomes and the development of precision medicine strategies for RA.

## Figures and Tables

**Table 1:** Recent studies of methylation and csDMARD response. Table Legend: MTX (methotrexate), HCQ (hydroxycholoroquine), SSZ (sulfasalazine), NK (Natural Killer cells), PBMCs (peripheral blood monocytes).

Study	Medication	Patients	Sample Type	Sample Timeline	Outcome	Methylation Assay
De Andres et al., 2015 [94]	MTX	19 RA (csDMARD naïve) 17 healthy controls	T, B, NK, monocytes, polymorphonuclear leukocytes	Baseline and 1 month	DAS28 at 6 months	Global methylation using mass spectrometry
Liebold et al., 2020 [65]	MTX, sarilumab, baricitinib or tofacitinib	45 RA patients 17 healthy controls	PBMCs	Baseline and 3 months	EULAR criteria [95] at 6 months	Global methylation using immunofluorescence staining
Gosselt et al., 2019 [96]	MTX, MTX + SSZ + HCQ + corticosteroids	181	Leukocytes	Baseline and 3 months	EULAR criteria at 3 months	Global methylation using mass spectrometry
Glossop et al., 2017 [97]	MTX, SSZ and HCQ	46 RA (csDMARD naïve)	T-cells	Baseline	EULAR criteria at 6 months	Illumina HumanMethylation450 BeadChip Array
Nair et al., 2017 [98]	MTX	72 RA (36 GR, 36 NR)	Whole blood	Baseline and 4 weeks	EULAR criteria at 6 months	Illumina HumanMethylation450 BeadChip Array
Gosselt et al., 2021 [99]	MTX, MTX + corticosteroidsMTX+SSZ/HCQ	68 RA (csDMARD naive)	PBMCs	Baseline and at 3 months	DAS28 at 3 months	Illumina Human Methylation EPIC array
Guderud et al., 2021 [70]	MTX	11 RA (csDMARD naïve) 18 RA (MTX treated) 7 healthy controls	CD4+ T-cells	Baseline	N/A	Representation bisulfite sequencing

**Table 2:** Studies of methylation and bDMARD response. Table Legend: ADA (adalimumab), CTZ (certolizumab), ETN (etanercept), GOL (golimumab), IFX (infliximab), GR (good responder), NR (non-responder).

Study	Medication	Patients	Sample Type	Sample Timeline	Outcome	Methylation Assay
Plant et al., 2017 [101]	ETN	72 (36 GR/36 NR)	Whole blood	Baseline	EULAR criteria at 3 months	Illumina HumanMethylation450 BeadChip Array
Julia et al., 2022 [102]	TNFi (ADA, CTZ, ETN, GOL, IFX)	62 RA (discovery cohort) 60 RA (validation cohort)	Whole blood	Baseline and 3 months	EULAR criteria at 3 months	Illumina Human Methylation EPIC array
Tao et al., 2021 [103]	ADA, ETN monotherapy	80 RA	PBMCs, monocytes, CD4+ T-cells	Baseline	EULAR criteria at 6 months	Illumina Human Methylation EPIC array

## Data Availability

Not applicable.
